# Identification and Progression of Heart Disease Risk Factors in Diabetic Patients from Longitudinal Electronic Health Records

**DOI:** 10.1155/2015/636371

**Published:** 2015-08-25

**Authors:** Jitendra Jonnagaddala, Siaw-Teng Liaw, Pradeep Ray, Manish Kumar, Hong-Jie Dai, Chien-Yeh Hsu

**Affiliations:** ^1^School of Public Health and Community Medicine, University of New South Wales, Sydney, NSW 2052, Australia; ^2^Asia-Pacific Ubiquitous Healthcare Research Centre, University of New South Wales, Sydney, NSW 2052, Australia; ^3^Prince of Wales Clinical School, University of New South Wales, Sydney, NSW 2052, Australia; ^4^Department of Computer Science and Information Engineering, National Taitung University, Taitung 95092, Taiwan; ^5^Master Program in Global Health and Development, College of Public Health and Nutrition, Taipei Medical University, Taipei 11042, Taiwan; ^6^Department of Information Management, National Taipei University of Nursing and Health Sciences, Taipei 11219, Taiwan

## Abstract

Heart disease is the leading cause of death worldwide. Therefore, assessing the risk of its occurrence is a crucial step in predicting serious cardiac events. Identifying heart disease risk factors and tracking their progression is a preliminary step in heart disease risk assessment. A large number of studies have reported the use of risk factor data collected prospectively. Electronic health record systems are a great resource of the required risk factor data. Unfortunately, most of the valuable information on risk factor data is buried in the form of unstructured clinical notes in electronic health records. In this study, we present an information extraction system to extract related information on heart disease risk factors from unstructured clinical notes using a hybrid approach. The hybrid approach employs both machine learning and rule-based clinical text mining techniques. The developed system achieved an overall microaveraged *F*-score of 0.8302.

## 1. Introduction

Heart disease is a collective term for conditions that affect the heart. Heart disease often leads to serious cardiovascular events such as heart attacks and stroke. It has been observed to be the leading cause of death worldwide in both men and women and has become a major burden on national healthcare expenditures around the world [1, 2]. Assessment of the risk of heart disease is very crucial in finding opportunities for prevention. Identifying and tracking the progression of heart disease risk factors are the basic steps in heart disease risk assessment. A few examples of heart disease risk factors are diabetes, coronary artery disease (CAD), hypertension, hyperlipidemia, obesity, medications, smoking history, and family history of premature CAD. Data for these risk factors are often specifically collected for the purpose of studies assessing the risk of heart disease.

The healthcare sector observed a rapid adoption of electronic health record (EHR) systems in the past decade. The primary purpose of EHR systems is to collect, store, and exchange patient data. EHRs are rich sources of valuable patient data such as comorbidities, medication history, social history, and family history. Data gathered from EHRs can be used as an alternative for data collected from studies specifically designed for heart disease risk assessment. However, most of these valuable patient data are buried in the form of unstructured format in EHRs [3, 4]. Manually extracting these unstructured data from EHRs can be very expensive and time consuming. Extracting unstructured data required for risk assessments can be automatically done using clinical text mining. This involves two major subtasks: identifying risk factors and tracking the progression of the disease. Automatic extraction of these heart disease risk factor data involves developing a highly specified system and may not be suitable for extracting risk factors for other diseases without necessary changes.

Recently, a great increase in information extraction (IE) systems catered for the clinical domain has been observed. There are various open source IE systems available to extract information from unstructured EHRs [5–12]. However, the types of heart disease risk factor information with temporality that can be extracted from these IE systems are limited. cTAKES is an open source IE system useful in extracting disease disorders, medications, symptoms, and anatomical locations [11]. HITEx is another clinical IE system based on the GATE framework capable of extracting disease disorders, medications, and smoking status [13]. MedEx is another IE system useful in extracting medication information [12]. TEMPTING, on the other hand, is an IE system capable of extracting temporal relations useful in tracking the progression of the disease from patient discharge summaries [5]. Byrd et al. developed a hybrid IE system to extract Framingham diagnostic criteria for heart failure with relevant disease progression information [14]. Another example is the rule-based FRSSystem capable of extracting Framingham risk factors used for predicting the risk of CAD [15]. Jonnagaddala et al. developed a machine learning-based IE system to identify disease disorder mentions [8]. The mentioned IE systems can be reused to identify heart disease risk factors but often require customization or addition of new modules. Savova et al. built a machine learning-based smoking classification module for cTAKES [16]. Goryachev et al. developed a module for HITEx to extract family history related information. None of these systems can identify a comprehensive number of heart disease risk factors that can be used for risk assessment.

In this study, we present an IE system capable of extracting unstructured data from EHRs. This is specifically developed for the purpose of identifying and tracking the progression of heart disease risk factors in diabetic patients. The system developed in this study is an extension to our baseline system which was developed as part of our participation in the 2014 i2b2/UTHealth shared tasks [17, 18]. The developed system performs risk factor concept recognition and assigns relevant time attributes to the recognized risk factors on longitudinal EHRs. The heart disease risk factors recognized by the system are diabetes, coronary artery disease (CAD), hypertension, hyperlipidemia, smoking status, obesity status, family history of premature CAD, and medications. The system extracts the above-mentioned heart disease risk factors and assigns an indicator attribute and a time attribute, if applicable. The system is a hybrid system with both rule-based and machine learning components. The evaluation of the system shows that it achieved an overall microaveraged *F*-score of 0.8302.

## 2. Materials and Methods

### 2.1. Dataset

**Figure 1:**
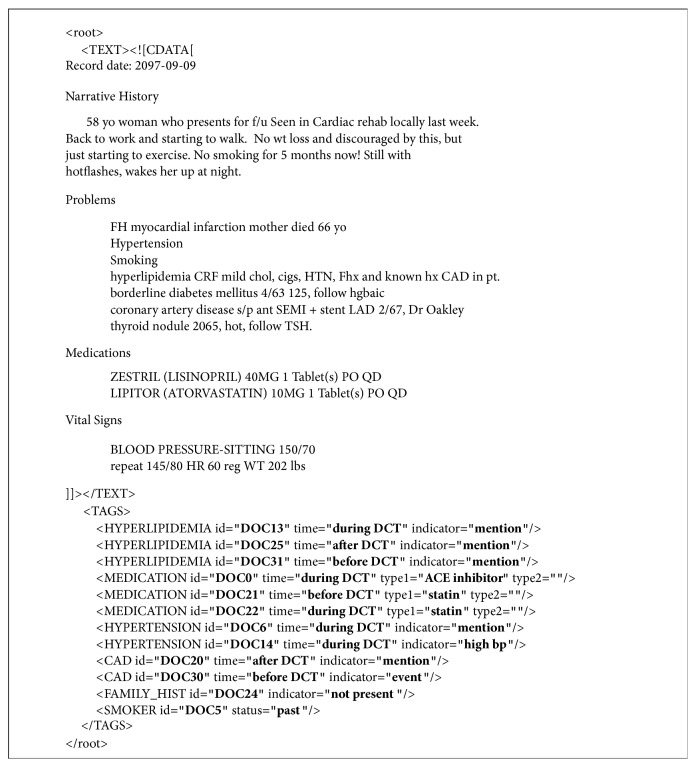
Sample EHR with annotations of heart disease risk factors.

The authors used the 2014 i2b2/UTHealth shared task 2 dataset in this study [18]. The dataset is a collection of unstructured longitudinal EHRs of diabetic patients provided by Partners Healthcare, USA. The EHRs are deidentified and annotated according to the guidelines. The annotations included heart disease risk factors and information of disease progression [19]. Gold standard annotations for this dataset were also available to evaluate the developed IE system. The dataset included 1304 unstructured EHRs (from here on referred to as records) from 297 patients divided into three sets: training set 1, training set 2, and test set. Training set 1 and training set 2 included 521 and 269 records, respectively, while the test set had 514 records. The dataset was also stratified into three different cohorts of diabetic patients: patients who had CAD, patients who develop CAD, and patients who did not develop CAD over a period of time [15]. Presence of heart risk factors and progression of the disease were defined in the form of risk factor, indicator attribute, and time attribute in the dataset. An overview of risk factors and their corresponding attributes is presented in Table 1. A sample (modified) EHR from the dataset is also illustrated in Figure 1. Each risk factor tag excluding family history and smoking history had time attribute that can take values, before document creation time (DCT), during DCT, and after DCT. The time attribute defines when a risk factor is known to have existed. The indicator attribute defines whether the identified risk factor is a mention, test, or lab value.

### 2.2. System Description

**Figure 2:**
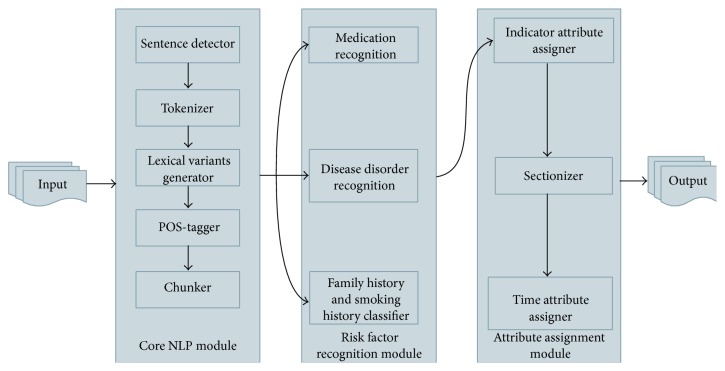
Overview of heart disease risk factor information extraction system.

The heart disease risk factors system (HDRFSystem) in its current form includes three modules (i) core NLP module, (ii) risk factor recognition module, and (iii) attribute assignment module (Figure 2). The core NLP module identifies sentence boundaries (sentence detector), breaks sentences into tokens (tokenizer), assigns part of speech tags (POS-tagger), and identifies noun phrases (chunker). The core NLP module adopted components from the OpenNLP package (v1.5.3) available at https://opennlp.apache.org/. Processed information from the core NLP module is then passed to the risk factor recognition module where medications, disease disorder mentions, family history, and smoking history are identified. The risk factor recognition module is responsible for identifying all the heart disease risk factors. All the identified risk factors (except family history and smoking history) were then assigned indicator and time attributes by the components in the attribute assignment module. The components of the risk factor recognition module and the time attribute assignment module are explained in more detail in the following sections.

#### 2.2.1. Medication Recognition

This component was used for identification of medications and is based on MetaMap [20, 21]. The noun phrase chunks identified by the chunker component in the core NLP module were passed to MetaMap. The component was configured to use MetaMap with UMLS2013AB as the knowledge source and USAbase as the data version and strict data model. For identifying medications, the component was restricted to use RxNorm terminology with a candidate score of 1000.

#### 2.2.2. Disease Disorder Recognition

This component identifies the mentions of hypertension, hyperlipidemia, CAD, and obesity using MetaMap. To identify disease disorder MetaMap was configured to use SNOMEDCT_US terminology as source with a candidate score of 1000. Rules were developed for finding lab values such as blood pressure values, HDL count, and glucose level. For example, this component can identify BP value from text such as “BP: 158/72,” “blood pressure 149/96,” or “blood pressure elevated at 188/92.” Similarly, the authors developed rules to identify lipid levels (e.g., lipid levels: total cholesterol 164, TG 145, HDL 33, and LDL 102) and other blood tests (e.g., BUN is 27, creatinine is 4.7, and glucose is 79). Once values were identified, they were filtered out based on the levels mentioned in the annotation guidelines [19]. This component also filters out irrelevant disease disorders which are not considered as heart disease risk factors based on the rules using UMLS CUI. Furthermore, a custom-built dictionary was used to find abbreviation mentions. For example,* DM2, DM Type II*, and* DMII* refer to diabetes type 2. The rules in this component were implemented using Apache UIMA Ruta framework (https://uima.apache.org/ruta.html).

#### 2.2.3. Family History and Smoking History Classifier

A rule-based classifier was employed to identify family history of premature CAD. This rule-based classifier identifies sentences containing CAD mentions that also has mention of familial relationships. More rules were applied to check whether the relative died prematurely (age < 55) due to CAD. If there are no such sentences in the document, then the document is simply classified as unknown for family history of premature CAD. In the dataset, smoking history is classified at document level using five classes: “current,” “past,” “never,” “ever,” and “unknown.” We developed Naïve Bayes algorithm-based supervised learning classifier to identify smoking history in conjunction with a few rules [22]. The Naïve Bayes classifier model was built using features illustrated in Table 2. Furthermore, we evaluated and selected features which were highly correlated with the classifier's predicative performance [23]. The smoking history classifier identifies smoking history by classifying each sentence. If multiple instances of smoking sentences were identified, rules were applied to select one. The training dataset was used to build a custom dictionary of smoking terms such as smoker, tobacco, and packs per year that can be used to identify sentences containing any mention of smoking history. Using this custom-built dictionary, sentences for smoking history mention were identified and were further classified into three classes, namely, “current,” “past,” and “never.” During the development of classifier, it was noticed that less than 1% of the records in the dataset belong to “ever” class. To improve classifier performance, “ever” class was completely ignored. If no mention of smoking terms was found in the document, then that document was simply classified as “unknown” for smoking history.

#### 2.2.4. Indicator Attribute Assigner

The indicator attribute assigner takes input from the risk factor recognition module and assigns appropriate indicator attribute based on dataset annotation guidelines [19]. This component was developed by implementing various rules using Apache UIMA Ruta framework. The rules consider factors like how the risk factor was recognized and what the annotations made by the risk factor module were. For example, if a record contains text such as “type 1 diabetes,” the diabetes risk factor is recognized by the disease disorder component, and it is assigned with “mention” tag for the indicator attribute. Similarly, using the same rules, if the diabetes risk factor was recognized by A1c test values, it is assigned with “high A1c” indicator attribute. Medication type was assigned using a custom-built dictionary built from the training datasets using Wikipedia. The final dictionary file contained medications generic names and categories they belong to. In total, there were 474 medications in 21 categories (related to heart disease risk factors). Overall, we developed 26 rules to assign risk factor indicator attribute.

#### 2.2.5. Sectionizer

Most of the EHRs in the dataset included section headings. The section headings information was useful in identifying family history and medication risk factors [17, 24]. At the same time, the same information was used as a feature for assigning time attribute to identified risk factors. For example, medications mentioned under section heading “medications” or “medications on admission” will always have a time attribute as before DCT, after DCT, and during DCT. Thus, we developed a conditional random field (CRF) based machine learning classifier to identify section information using features illustrated in Table 2 [25]. The classifier classified a sentence to either “section heading” or “section heading with text” or “text.” Section heading class was assigned if the sentence contains only a section heading (e.g., current medications). Section heading with text class was assigned if the sentence contains section heading with text (e.g., record date: 2073-12-14). Text class was assigned if the sentence contains text only and does not include any section headings (e.g., “s/p XRT to esophagus”).

#### 2.2.6. Time Attribute Assigner

This component assigns time attribute for each of the risk factors identified by earlier components. Similar to the smoking history classifier, a supervised learning classifier based on Naïve Bayes algorithm was developed with addition of a few rules to complete the task [22]. We used risk factor phrases annotated by risk factor recognition module to train the model with features shown in Table 2. Each phrase was classified into either one of the four classes shown in Table 2. When a phrase is classified as continuing in the output, we assigned all three time attributes, before DCT, after DCT, and during DCT, as per annotation guidelines [19].

## 3. Results

The HDFRSystem was evaluated using macro- and microaveraged precision, recall, and *F*-score [26]. An evaluation script provided with the dataset was used to calculate performance scores. The evaluation script is capable of reporting system performance at many levels including specific risk factors by indicator attribute and time attribute. The evaluation metrics are explained in more detail elsewhere [19]. The developed system achieved an overall microaveraged *F*-score of 0.8302 on the test set. Performance of the developed system on the test set categorized by indicator attribute is presented in Table 3. Every mention indicator attribute outperformed other indicator attributes, suggesting that our methods were effective in identifying risk factor mentions but not so effective in inferring risk factors from lab values or tests. A number of indicator attributes were not recognized, specifically CAD test result, high glucose, high cholesterol, obesity, medications, amylin, antidiabetes, and waist circumference. The smoking history classifier also underperformed when compared to other risk factors by achieving 0.5 and 0.7265 microaveraged *F*-score for “current” and “never” indicator attributes, respectively.

We also present the system's performance categorized by time attribute in Table 4. Similar to the trend noticed in Table 3, CAD and medication risk factors underperformed when compared to other risk factors. CAD and medication risk factors achieved a lower recall and *F*-score when compared to overall risk factors for all three time attributes. In other words, our time attribute classifier did not perform well on assigning time attributes for CAD and medication risk factors. However, our time attribute classifier performed well for diabetes and hypertension achieving microaveraged *F*-scores of 0.9203 and 0.9464, respectively.

For comparison, we present the results of a cTAKES-based system (Table 5) versus the results we obtained (Table 6) using the test set [17]. Our system performed significantly better than the cTAKES-based system. The system developed in this study achieved a higher overall micro-*F*-score compared to the cTAKES-based system, 0.8302 versus 0.7151. Our methods outperformed the cTAKES-based system in all of the risk factors. Family history risk factor achieved the highest macro- and microaveraged *F*-score. All the risk factors achieved a microaveraged *F*-score above 0.80, except for CAD. Out of all the risk factors, CAD and medication risk factors achieved a lower macro- and microaveraged precision, recall, and *F*-score.

## 4. Discussion

We performed an extensive error analysis to understand our results in depth. Several interesting findings about the system and the dataset, in general, were observed. A few errors in the gold set annotations were also noticed. For example, the smoking history did not have annotations for all documents in the test set. Thus, being a document level classification problem, the evaluation metrics precision, recall, and *F*-score are not the same for smoking risk factor. Previously, we believed that our methods did not recognize amylin and antidiabetes as shown in Table 5. However, upon further inspection, the test set did not have any entities with amylin or antidiabetes. The training sets also had very few negated mentions of risk factors. Moreover, we found that there were very few instances (<1%) for “ever” smoking history class in the training set. This created an imbalance classification problem where the performance of classifier may not represent the full capabilities of the classifier [27]. So we simply removed “ever” class from our classification problem, to make it a balanced classification problem.

During the development of the system, we noticed that by employing simple rules we can drastically improve the performance of the system. As a result, we implemented rules in our components.  Table 7 summarizes the number of rules in each component with examples. From the results, it can be interpreted that for most of the risk factors MetaMap outperformed rule-based lab value extractor component. For example, diabetes mention had an *F*-score value of 0.8897 while diabetes A1c had 0.7808. This means that the rule-based lab value extractor was not as effective as we expected. The lab value extractor failed to recognize values represented as ranges. For example, the blood pressure value “120–130/88–92” was extracted as “130/88,” and as a result it was not detected as high blood pressure by our rules. One simple rule applied for time attribute assignment turned to be effective for diabetes, hypertension, hyperlipidemia, and obesity but not for CAD and medication. We believe that the poor performance of the time attribute assigner on the CAD and medication risk factors is due to usage of limited features.

We formulated the time attribute assignment problem as a classification problem and assigned one of the three time attributes to each of the risk factors identified by the system. Even though very few features like indicator type, identified token, and section information were used in building a Naïve Bayes model, the classifier performed well, achieving overall microaveraged *F*-scores of 0.8185, 0.814, and 0.8403 for before DCT, during DCT, and after DCT time attributes, respectively. At an individual risk factor level, the time attribute assigner component performed very well for hypertension. However, the performance for CAD risk factor was observed to be low. We believe this is due to the disease disorder component, which failed to recognize CAD risk factor effectively. The performance of the time attribute assigner component can be further improved by adding context and negation based features [28].

We also noticed that the dataset included numerous abbreviated disease and medication mentions. ASA (acetyl salicylic acid), NTG (nitroglycerin), TNG (trinitroglycerin), DM (diabetes mellitus), and HTN (hypertension) are few examples to mention. Even though a custom abbreviations dictionary was prepared by the authors using the training sets, the test set included several other abbreviations which were not included in the custom dictionary list. Employing a more sophisticated abbreviation handling technique which is not specific to a particular dataset will improve the performance of system overall [29, 30]. Unified medical language system (UMLS) should be used to disambiguate abbreviations. We also found that there were a few misspelled mentions like pravastatin which was misspelled as “pravastain” and obese as “obeise.” Similar to abbreviations custom dictionary, another custom list was developed for misspellings from training sets. However, this approach is not generic and very specific to a given dataset; employing a generic spelling correction is necessary [31].

## 5. Conclusion

In summary, we described an approach to extract heart disease risk factors in diabetic patients from longitudinal unstructured EHRs. The approach was based on both rules and machine learning techniques. We also described an IE system developed using this approach followed by a comprehensive evaluation of the system. The system was developed using one dataset and might not perform well on other datasets, especially with the rules that were developed. The limitations of the system include issues in lab value extractor and absence of negation and context aware components. In the future, we would like to improve the performance of sectionizer component and also build negation components into the system. We also would like to explore a more sophisticated method to disambiguate abbreviations and handle misspellings effectively. The developed system is available for free at https://github.com/TCRNBioinformatics/HDRFSystem.

## Figures and Tables

**Table 1:** Overview of risk factors, indicator attribute, and time attribute.

Risk factor	Indicator attribute	Time attribute
CAD	Mention, event, test result, and symptom	Before DCT, during DCT, and after DCT

Diabetes	Mention, high A1c, and high glucose	Before DCT, during DCT, and after DCT

Family history	Present, not present	Not applicable

Hyperlipidemia	Mention, high cholesterol, and high LDL	Before DCT, during DCT, and after DCT

Hypertension	Mention, high blood pressure	Before DCT, during DCT, and after DCT

Medication	ACE inhibitors, ACE inhibitors ARBs, amylin, antidiabetes medications, aspirin, beta-blockers, calcium-channel blockers, DPP-4 inhibitors, ezetimibe, fibrates, GLP-1 agonists, insulin, meglitinides, metformin, niacin, nitrates, obesity, statins, sulfonylureas, thiazide diuretics, thiazolidinediones, and thienopyridines	Before DCT, during DCT, and after DCT

Obesity	Mention, BMI, and waist circumference	Before DCT, during DCT, and after DCT

Smoking history	Current, past, ever, never, and unknown	Not applicable

**Table 2:** Features used by smoking history, sectionizer, and time attribute assigner classifiers.

Component	Classification	Classifier	Classes	List of features
Smoking history	Sentence level	Naïve Bayes	Current, past, and never	Bag of words, POS tags

Sectionizer	Sentence level	Conditional random fields	Section heading, section heading with text, and text	First word uppercased, all words uppercased, all words lowercased, dictionary match, first word, second word, previous sentence features, next sentence features, full stop, and containing colon

Time attribute assigner	Phrase level	Naïve Bayes	Before DCT, during DCT, after DCT, and continuing	Identified risk factor spans, previous word, previous word POS tag, next word, next word POS tag, section information, and indicator attribute

**Table 3:** Performance on test set by indicator attributes.

Risk factor	Macroaveraged			Microaveraged		
	Precision	Recall	*F*-score	Precision	Recall	*F*-score
CAD						
Mention	0.3346	0.3405	0.3375	0.5029	1.0000	0.6693
Event	0.1148	0.1138	0.1143	0.8806	0.4245	0.5728
Test result	0.0000	0.0000	0.0000	0.0000	0.0000	0.0000
Symptom	0.0000	0.0000	0.0000	0.0000	0.0000	0.0000
Diabetes						
Mention	0.6887	0.6907	0.6897	0.9219	0.9972	0.9581
High A1c	0.1109	0.106	0.1084	0.8906	0.6951	0.7808
High glucose	0.0000	0.0000	0.0000	0.0000	0.0000	0.0000
Family history						
Present	0.0097	0.0097	0.0097	1.0000	0.2632	0.4167
Not present	0.9630	0.9630	0.9630	0.9725	1.0000	0.9861
Hyperlipidemia						
Mention	0.4436	0.4436	0.4436	0.8444	0.962	0.8994
High cholesterol	0.0000	0.0000	0.0000	0.0000	0.0000	0.0000
High LDL	0.0331	0.0331	0.0331	0.7391	0.5862	0.6538
Hypertension						
Mention	0.7062	0.7101	0.7082	0.9553	0.9918	0.9732
High blood pressure	0.2996	0.2889	0.2942	0.4858	0.7897	0.6016
Medication						
ACE inhibitors	0.3320	0.3482	0.3399	0.8797	0.8325	0.8555
ACE inhibitors ARBs	0.1096	0.1128	0.1112	0.8667	0.8756	0.8711
Amylin	0.0000	0.0000	0.0000	0.0000	0.0000	0.0000
Antidiabetes Medications	0.0000	0.0000	0.0000	0.0000	0.0000	0.0000
Aspirin	0.3709	0.3930	0.3817	0.9079	0.7168	0.8011
Beta-blockers	0.3891	0.4047	0.3967	0.9302	0.7186	0.8108
Calcium-channel blockers	0.2010	0.2160	0.2082	0.9064	0.8052	0.8528
DPP-4 inhibitors	0.0039	0.0039	0.0039	1.0000	1.0000	1.0000
Ezetimibe	0.0214	0.0253	0.0232	0.6471	0.9167	0.7586
Fibrates	0.0506	0.05447	0.0525	0.8966	0.8667	0.8814
GLP-1 agonists	0.0000	0.0000	0.0000	0.0000	0.0000	0.0000
Insulin	0.1790	0.1887	0.1837	0.8598	0.6987	0.7709
Meglitinides	0.0000	0.0000	0.0000	0.0000	0.0000	0.0000
Metformin	0.2069	0.2228	0.2145	0.8439	0.8598	0.8518
Niacin	0.0123	0.0175	0.0144	0.4524	0.7600	0.5672
Nitrates	0.1031	0.1148	0.1086	0.803	0.5867	0.6780
Obesity	0.0000	0.0000	0.0000	0.0000	0.0000	0.0000
Statins	0.4617	0.4786	0.4700	0.9199	0.8715	0.8950
Sulfonylureas	0.1518	0.1595	0.1555	0.9286	0.8125	0.8667
Thiazide Diuretics	0.1226	0.1376	0.1297	0.3058	0.7441	0.4335
Thiazolidinediones	0.0396	0.04475	0.0420	0.8841	1.0000	0.9385
Thienopyridines	0.1543	0.1673	0.1606	0.8914	0.8380	0.8639
Obesity						
Mention	0.1589	0.1693	0.1639	0.7632	1.0000	0.8657
BMI	0.0136	0.0123	0.0129	1.0000	0.4118	0.5833
Waist circumference	0.0000	0.0000	0.0000	0.0000	0.0000	0.0000
Smoking history						
Current	0.0234	0.0234	0.0234	0.8000	0.3636	0.5000
Past	0.1479	0.1479	0.1479	0.8636	0.6726	0.7562
Ever	0.0000	0.0000	0.0000	0.0000	0.0000	0.0000
Never	0.1576	0.1576	0.1576	0.7864	0.6750	0.7265
Unknown	0.4728	0.4728	0.4728	0.7890	1.0000	0.8820

**Table 4:** Performance on test set by time attributes.

Risk factor	Macroaveraged			Microaveraged		
	Precision	Recall	*F*-score	Precision	Recall	*F*-score
CAD						
Before DCT	0.3434	0.2628	0.2977	0.5599	0.5827	0.5711
During DCT	0.3405	0.3176	0.3286	0.5117	0.8102	0.6272
After DCT	0.3327	0.3288	0.3307	0.5000	0.9771	0.6615
Diabetes						
Before DCT	0.6848	0.6683	0.6765	0.9152	0.9255	0.9203
During DCT	0.6907	0.6699	0.6801	0.9245	0.9293	0.9269
After DCT	0.6887	0.6887	0.6887	0.9219	0.9972	0.9581
Hyperlipidemia						
Before DCT	0.4504	0.4429	0.4466	0.8419	0.9007	0.8703
During DCT	0.4426	0.4407	0.4416	0.8382	0.9421	0.8872
After DCT	0.4436	0.4436	0.4436	0.8444	0.962	0.8994
Hypertension						
Before DCT	0.7043	0.6868	0.6954	0.9526	0.9403	0.9464
During DCT	0.644	0.7364	0.6871	0.7432	0.9557	0.8362
After DCT	0.7062	0.7062	0.7062	0.9553	0.9918	0.9732
Medication						
Before DCT	0.6768	0.6600	0.6683	0.8332	0.7923	0.8122
During DCT	0.6613	0.6519	0.6565	0.8095	0.7858	0.7975
After DCT	0.6729	0.6648	0.6688	0.8200	0.7943	0.8069
Obesity						
Before DCT	0.1537	0.1518	0.1527	0.7383	0.9753	0.8404
During DCT	0.1693	0.1634	0.1663	0.8246	0.9400	0.8785
After DCT	0.1537	0.1518	0.1527	0.7383	0.9753	0.8404
All risk factors						
Before DCT	0.7727	0.7881	0.7803	0.8224	0.8146	0.8185
During DCT	0.7463	0.8187	0.7808	0.7835	0.8470	0.8140
After DCT	0.7706	0.8321	0.8002	0.8136	0.8688	0.8403

**Table 5:** Performance of baseline system on test set.

Risk factor	Macroaveraged			Microaveraged		
	Precision	Recall	*F*-score	Precision	Recall	*F*-score
CAD	0.2135	0.2311	0.2220	0.6652	0.5599	0.6080
Diabetes	0.6576	0.6745	0.6660	0.8692	0.9517	0.9086
Family history	0.9689	0.9689	0.9689	0.9689	0.9689	0.9689
Hyperlipidemia	0.4465	0.4412	0.4439	0.8434	0.9254	0.8825
Hypertension	0.3429	0.4833	0.4012	0.5579	0.6148	0.5850
Medication	0.5486	0.6534	0.5964	0.6227	0.7409	0.6767
Obesity	0.1402	0.1419	0.141	0.8447	0.8511	0.8479
Smoking history	0.6284	0.6284	0.6284	0.6284	0.6309	0.6296

Overall	0.6954	0.7634	0.7278	0.6779	0.7566	0.7151

**Table 6:** Performance of HDRFSystem on test set.

Risk factor	Macroaveraged			Microaveraged		
	Precision	Recall	*F*-score	Precision	Recall	*F*-score
CAD	0.3455	0.2985	0.3203	0.5261	0.7334	0.6127
Diabetes	0.6876	0.6724	0.6799	0.9202	0.9483	0.9341
Family history	0.9728	0.9728	0.9728	0.9728	0.9728	0.9728
Hyperlipidemia	0.4504	0.4451	0.4477	0.8415	0.9334	0.8851
Hypertension	0.6970	0.7375	0.7166	0.8531	0.9613	0.9040
Medication	0.6703	0.6731	0.6717	0.8209	0.7908	0.8056
Obesity	0.1589	0.1652	0.1620	0.7683	0.9618	0.8542
Smoking history	0.8113	0.8113	0.8113	0.8113	0.8145	0.8129

Overall	0.8053	0.8515	**0.8277**	0.8138	0.8472	**0.8302**

**Table 7:** Examples of rules used in HDRFSystem components.

Component	Number of rules	Examples
Medication recognition	12	If the medication identified by MetaMap is from RxNorm terminology, assign risk factor with identified medication name.
		If the medications identified by MetaMap include abbreviations from custom abbreviations dictionary, assign medication risk factor with full medication name.

Disease disorder recognition	22	If the disease identified by MetaMap is from SNOMED CT terminology and is either CAD or obesity or diabetes or hypertension or hyperlipidemia, assign risk factor with identified disease name.
		If annotated text is identified by blood pressure lab value extractor and diastolic >90 or systolic >140, assign risk factor = “hypertension.”

Family history	05	If a sentence contains “cad” or “coronary artery disease” and contains “father,” “mother,” or brother, assign sentence as family history sentence.
		If family history sentence contains age of death and age <45, assign family history = “present” or else “unknown.”

Smoking history	07	If a sentence contains terms from custom smoking terms dictionary, assign sentence as smoking history sentence.
		If document does not contain smoking terms, assign smoking history = “unknown.”

Sectionizer	04	If a sentence is classified as “text” but contains terms from custom section headings dictionary, assign label “section heading.”
		If a sentence is classified as “section heading with text” and contains “:”, extract text before “:” to obtain section information.

Indicator attribute assigner	26	If annotated text is identified by MetaMap, assign attribute = “mention.”
		If annotated text is identified by blood pressure lab value extractor and diastolic >140 or systolic >90, assign indicator attribute = “high BP.”

Time attribute assigner	01	If time attribute assigner assigned class is “continuing,” assign time attributes = “before DCT,” “after DCT,” and “during DCT.”
